# The Prevalence and Clinical Phenotypes of Cluster Headache in Relation with Latitude

**DOI:** 10.1007/s11916-024-01229-3

**Published:** 2024-03-05

**Authors:** Yi-Chia Liaw, Shih-Pin Chen, Shuu-Jiun Wang

**Affiliations:** 1 Department of Neurology, Neurological Institute, Taipei Veterans General Hospital, Taipei, Taiwan; 2 School of Medicine, College of Medicine, National Yang Ming Chiao Tung University, Taipei, Taiwan; 3 Brain Research Center, National Yang Ming Chiao Tung University, Taipei, Taiwan; 4 Institute of Clinical Medicine, National Yang Ming Chiao Tung University, Taipei, Taiwan; 5 Division of Translational Research, Department of Medical Research, Taipei Veterans General Hospital, Taipei, Taiwan

**Keywords:** Cluster headache, Lifetime prevalence, 1-year prevalence, Cranial autonomic symptoms, Latitude

## Abstract

**Purpose of Review:**

Previous studies have indicated a possible link between the prevalence of cluster headache (CH) and sunlight exposure. However, this theory has yet to be tested systemically. In this article, we aim to examine how latitude affects the prevalence and phenotypes of CH.

**Recent Findings:**

To our knowledge, there is by far no article describing the effect of latitude on disease phenotype; thus, we performed a literature review. We noted positive effects of latitude on 1-year prevalence, the proportion of chronic CH, and the proportion of miosis and/or ptosis.

**Summary:**

Latitude may affect the phenotypic presentations of cluster headache, probably partially mediated via temperature and sunlight variations. Still, other factors, such as environmental exposure to smoking and the genetic difference between the Eastern and Western populations, may participate in the pathogenesis and clinical manifestations of CH.

**Supplementary Information:**

The online version contains supplementary material available at 10.1007/s11916-024-01229-3.

## Introduction

Cluster headache (CH) is a primary headache disorder listed under the category of trigeminal autonomic cephalalgia (TAC), as defined by the International Classification Headache Disorders, 3rd edition (ICHD-3) [1]. CH is characterized by severe pain and the featured cranial autonomic symptoms (CAS), including unilateral lacrimation, conjunctival injection, rhinorrhea, nasal congestion, eyelid edema, forehead and facial sweating, miosis, and ptosis [1]. Though not listed among the diagnostic criteria, patients with CH present attacks periodically at a specific time within a day, i.e., the circadian rhythm, or within a year, i.e., the circannual rhythm. Thus, CH is also nicknamed the “alarm headache.” The hypothalamus, a structure that participates in day-night circadian regulation and sensing seasonal variation, has been proposed to play a central role in regulating the periodic presentations [2].

Latitude is a geographic coordinate related to several geometric measurements, such as temperature or sunlight variation [3]. One well-known example of how latitude affects a disease is through the study of multiple sclerosis, in which the disease is more prevalent among residents who live at a higher latitude [4]. Moreover, other autoimmune diseases, such as the latitude-dependent autoimmune diseases (LDADs), also showed a positive correlation between latitude and disease prevalence [5]. Among these disorders, a lowered vitamin D level at a higher latitude was commonly proposed to be one of the disease-causing mechanisms [4].

Previous studies have suggested a possible link between sunlight exposure and the prevalence of CH; moreover, seasonal preferences of bouts have also been reported in clinical studies [6]. Since latitude is the geographical coordinate that could affect sunlight variation [3], we hypothesized that there is a correlation between the clinical presentations of CH and latitude. Thus, we aimed to perform a literature review to decipher how geographic factors affect disease phenotype.

## Methods

### Literature Search

**Fig. 1:**
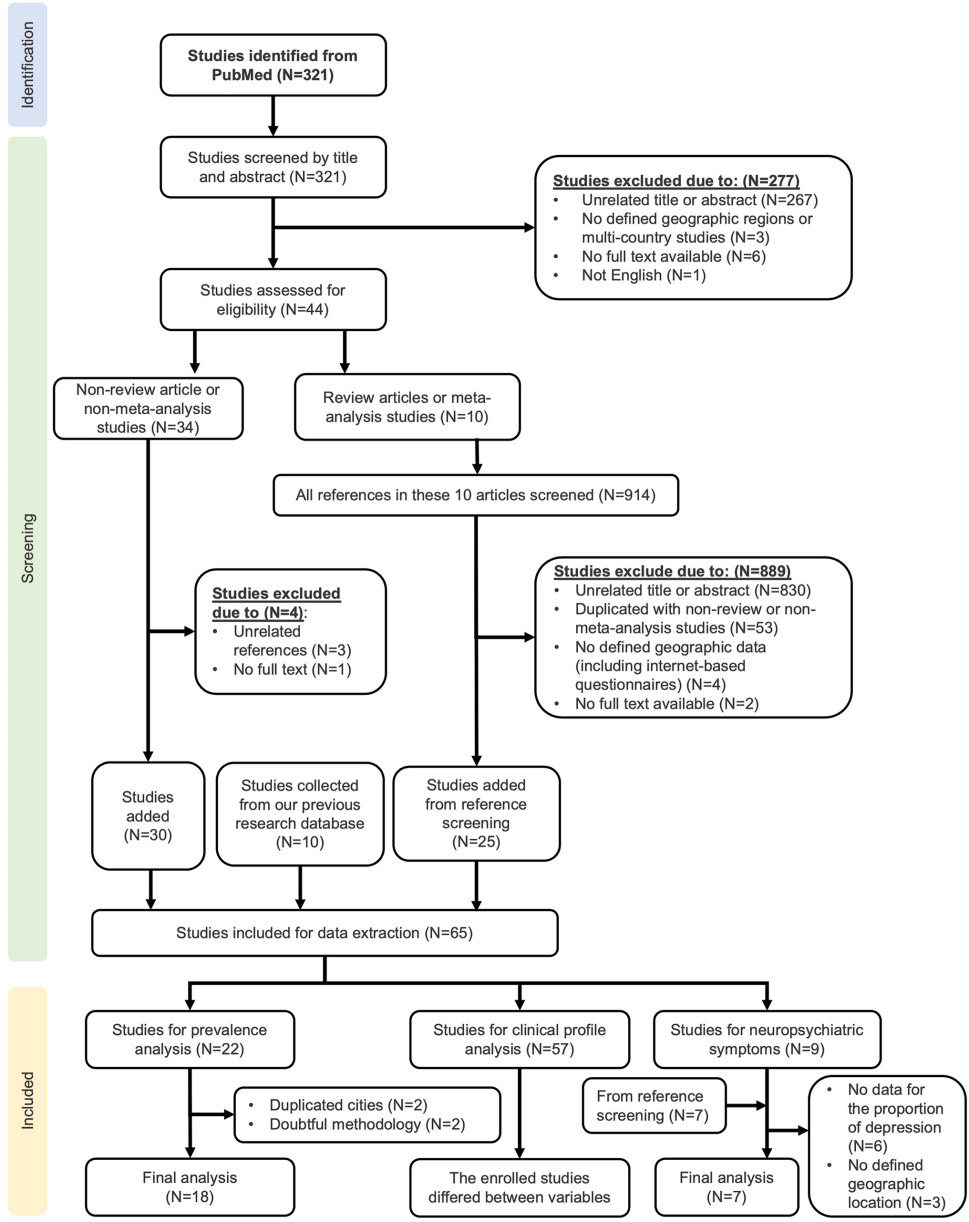
PRISMA flow chart

A literature review was performed with the PubMed database, and a selection diagram with PRISMA 2020 was utilized to identify eligible articles in our analysis (Fig. 1) [7]. In the searching step, the terms “Cluster headache” with Medical Subject Headings (MeSH) and “Epidemiology” were used (Query string: (Cluster headache [MeSH]) AND (Epidemiology)). The articles were searched up to November 24th, 2023, and there were no limitations to the year of publication or language of the publications.

A total of 321 studies were identified from PubMed, and their titles and abstracts were screened by one of the authors [Y.C.L.] independently. Two hundred and seventy-seven studies were excluded in the first step for the following reasons: (1) 267 studies with unrelated content, (2) three with no identified geographic regions or were multi-country studies that a specific geographic coordinate could not be defined, (3) six with no available full text, and (4) one without an English abstract. This resulted in 44 studies being assessed in the next step.

In the second step, a nested selection process was performed; that is, we searched the references from the selected articles to encompass a broader collection of the literature. We first divided the 44 articles into two main categories, by whether they were “meta-analysis or review article” (*N* = 10) or not (*N* = 34). We extracted all of the references from the ten articles assigned to the first category, and a second literature selection process was performed by screening their titles and abstracts. Initially, a total of 914 references were found. After 889 articles being excluded with specific reasons (Fig. 1), 25 articles survived this selection process. For the 34 articles listed in the second category, four articles were excluded for reasons specified in Fig. 1, and 30 articles survived. Lastly, we also searched our database from previous studies, and additional 10 studies, which are different from the previous 55 studies, were selected. Finally, a total of 65 studies were included for data extraction [6, 8–71].

### Prevalence Study

To investigate the relationship between disease prevalence and latitude, studies based on community population were selected. Since the bout frequency for most studies reported an average of 1 bout per year, and in Asian studies, the frequency is even lower [72], the actual headache prevalence might be underestimated if the study timeframe was short. Thus, we reported both the 1-year prevalence and lifetime prevalence separately among those studies based on their study designs or the content of questionnaires. Among the 22 studies recruited for data extraction, two were deemed ineligible for analysis and further excluded. A study from Addis Ababa, Ethiopia, reported 3 patients over 231 participants in the general population [37], which makes the lifetime prevalence of 1.2%. However, considering that this community encompassed only 400 households, and there were an average of four people in a household, the prevalence might be overestimated due to their close relationships. In another Swedish study [24], the prevalence was around 0.2% (48/31,750); however, since the population was based on a twin study, we excluded this article for the potential overestimation of the prevalence. Moreover, to deal with studies from the same community (two studies from San Marino [21, 33] and two from Stockholm, Sweden [8, 25]), only the studies with a larger population enrolled were kept [8, 21]. After selection, a total of 18 articles were enrolled for the prevalence study [8, 14, 16, 19, 21, 27, 30, 34–36, 38, 51–53, 55, 62, 67, 68].

### Clinical Presentations

To investigate the relationship between clinical presentations of CH and latitude, we collected the studies providing at least one clinical phenotype, and the phenotypes were extracted. Both community- and hospital-based studies were enrolled. We enrolled all the studies, including episodic CH (eCH) or chronic CH (cCH). Moreover, if the studies provided only the data in subgroups (i.e., females and males) rather than the total population, we performed simple statistics to restore the data for the entire population by the patient numbers provided in the studies. A total of 57 articles were enrolled for final analysis [6, 9–15, 17, 18, 20–36, 38–50, 54, 56–71]. Since the reported clinical data differed from study to study, we did not exclude studies with duplicated cities in the initial step, and we enrolled as many studies as possible. However, during analysis, to avoid over-estimation, if a variable was available in multiple studies from the same city, the studies with the largest number of enrolled CH patients were kept due to a higher accuracy in a larger sample size. Therefore, the number of the studies differed in the analysis of each variable.

Regarding the analysis for cCH, since the selected articles spanned the timeframe before and after the implement of ICHD-3, the definition of cCH differed; thus, for the analysis regarding the proportion of cCH, we examined both conditions with (1) all studies enrolled, and (2) ICHD-3 subgroups alone. Moreover, since previous studies have suggested a strong correlation between smoking and cCH as well as a discrepancy between Eastern and Western populations, we adjusted both factors in the analysis of cCH [72–74].

In a previous study, the authors indicated a discrepancy of clinical presentations between Western and Eastern countries [72]. In this study, the studies from Western countries had a significantly higher latitude than those from Eastern countries. Therefore, for variables other than cCH, we adjusted this factor in the regression model for items that showed significance in Spearman’s correlation test. This study listed North and South American and Egyptian articles [67] as studies from Western countries.

### Neuropsychiatric Comorbidities

To examine the psychiatric comorbid conditions of cluster headaches, we investigated the contents of our collected literature. Though there were several studies describing psychological symptoms including depression, anxiety, and sleep disturbances, the methods and the assessment tools varied. After initial screening, 9 articles were enrolled [13, 17, 18, 26, 38, 42, 43, 49, 71]. An additional 7 articles were selected from reference screening [75–81]. Three studies were excluded due to no defined geographic boundaries [76, 80, 81]. For studies reporting depression, 7 reported the proportion of depression among the patients [13, 38, 42, 71, 75, 77, 79], and another four, all from Seoul, Korea, used the Patient Health Questionnaire-9 (PHQ-9) to assess the severity of depression [18, 26, 49, 78]. For anxiety, one reported the proportion of anxiety [18], one reported the proportion of “nervous disease” [13], and another four Korean studies reported the General Anxiety Disorder-7 (GAD-7) scores [18, 26, 49, 78] in participants. Two studies reported the total score of the Hospital Anxiety and Depression Scale (HADS) [17, 43]. Two studies reported suicidal attempts [17, 38]. Other assessment methods for psychological disorders included the Perceived Stress Scale 4 (PSS-4) in two studies [18, 26], EQ-5D in two studies [18, 26], and sleep conditions in one study [17]. Considering that (1) this manuscript is not a narrative review article, (2) the data of most psychological comorbidities assessments remained scarce, and (3) the studies reporting PHQ-9 and GAD-7 were located in the same city, we included only the proportion of depression in our final analysis [13, 38, 42, 71, 75, 77, 79].

### Latitude Definition

In this study, we defined the latitudes mostly from the communities’ locations. If a specific community was not indicated, the hospitals where the research were carried out were used as surrogates. For studies utilizing multicenter registries, we examined their study designs, and if > 50% of the patients could be located in a defined region, the studies were enrolled after a vote between the authors. However, for studies with a multi-nation design, the articles were omitted. Other conditions were discussed case by case within the authors whether the article should be kept or not. We defined the latitude of each location by an online website (https://www.latlong.net/).

### Statistics

The results were analyzed with R (R Core Team, 2019). For statistical analysis, non-parametric methods were carried out due to a relatively small sample size. For the correlation between latitude and the variables, Spearman’s correlation test was used. Linear regression was carried out to estimate the effect gradient on latitudinal change and to adjust for potential confounding factors. To test the differences between binary categories, such as the higher or lower latitude groups, the Wilcoxon rank sum test was applied. A significance level of all the statistics in our study was set at *p* value < 0.05 in a two-tailed test.

## Results

A total of 65 articles with available full text in English were qualified for analysis. Among these articles, 18 articles reported prevalence, and 57 reported clinical profiles. The average latitude from the Western studies was significantly higher than that in the Eastern studies (Western: median = 43.3°, range = 8.1°–67.3°; Eastern: median = 34.7°, range = 3.1°–39.9°, *p* = 0.007).

Nine articles with 1-year prevalence encompassed a latitude ranging from 3.1° to 59.3° (median = 35.7°) and nine articles with lifetime prevalence ranging from 40.3° to 63.4° (median = 43.9°) (Table 1). Both the 1-year prevalence (rho = 0.57, *p* = 0.121) and lifetime prevalence (rho = 0.35, *p* = 0.359) of CH showed positive trends with the latitude, but the statistical significances were not reached. Of note, if the significant outlier (800/100,000) [67] from Egypt on 1-year prevalence was removed, the correlation between latitude and 1-year prevalence becomes statistically significant (rho = 0.74, *p* = 0.046), and for every degree of increased latitude, the 1-year prevalence rate increased 1.69/100000 (*p* = 0.038). There was no significant East–West difference for 1-year prevalence (*p* = 0.643).

**Table 1:** One-year, lifetime prevalence and latitude

Ref	Country	City	Total cases (*N*)	CH cases (*N*)	Latitude	Prevalence^a^
**1-year prevalence**						
[8]	Sweden	Stockholm	5,945,895	3,240	59.33	54.5
[36]	Germany	Dortmund	1312	2	51.51	152.4
[34]	Germany	Essen	3336	4	51.46	119.9
[55]	France	Limousin (France)	1563	1	45.81	64
[68]	Iran	Tehran	3655	3	35.69	82.1
[67]	Egypt	Fayoum	2375	19	29.39	800
[27]	Brazil	Barbacena	36,145	15	21.23	41.5
[19]	Ethiopia	Butajira town	15,500	5	8.12	32.3
[51]	Malaysia	Kuala Lumpur	595	0	3.14	0.0
**Lifetime prevalence**						
[38]	Norway	Trondheim	3,892,260	1,891	63.43	48.6
[14]	Norway	Vaga	1838	7	61.88	380.8
[53]	Denmark	Copenhagen	740	1	55.68	135.1
[30]	Italy	Parma	7522	21	44.80	279.2
[21]	San Marino	San Marino	26,628	15	43.94	56.3
[35]	USA	Rochester	9837	26	43.16	264.3
[16]	Georgia	Tbilisi and Kakheti	1145	1	41.69	87.3
[62]	Portugal	Porto	2008	2	41.16	99.6
[52]	Greece	Athos (Greece)	449	0	40.27	0.0

^a^Per 100,000 population

A total of 40 studies reported the proportion of cCH, and the latitudes of these studies ranged from 25.0° to 61.9° (median = 44.8°) (Table 2). After removal of duplicate cities, there was a significantly positive correlation between the proportion of cCH and latitude (rho = 0.54, *p* < 0.001) in 19 cities, and for every one degree increase in latitude, the proportion of cCH increased 0.5% (*p* = 0.0003). If both smoking and East–West category were adjusted, there were no significant effect for latitude (*p* = 0.134), smoking rate (*p* = 0.365), or East–West category (*p* = 0.058). Among the 9 studies utilizing ICHD-3 for diagnosing cCH, the correlation between the proportion of cCH and latitude remained significantly positive (rho = 0.76, *p* = 0.007), and for every one degree increase in latitude, the proportion of cCH increased 0.7% (*p* = 0.007). After adjustment, there were no significant effect for latitude (*p* = 0.503), smoking rate (*p* = 0.536), nor East–West category (*p* = 0.174).

**Table 2:** Demographic profiles, clinical features and latitude

Phenotypes		Latitude		rho	*P* value	References
Types	Median (range)	N	Median (range)			
**Demographic profiles**						
1-year prevalence	64.0 (0–800)	8	35.7 (3.1–59.3)	0.74	0.046	[8, 19, 27, 34, 36, 51, 55, 68]
lifetime prevalences	99.6 (0–380)	9	43.9 (40.3–63.4)	0.35	0.359	[14, 16, 21, 30, 35, 38, 52, 53, 62]
All cCH (%)	12.5 (0–36.8)	21	39.9 (25.0–61.9)	0.54	< 0.001	[9, 11–15, 17, 18, 22, 31–33, 36, 39, 43, 45–47, 60, 61, 70]
cCH by ICHD-3 (%)	12.5 (1.3–35.9)	9	41.9 (25.0–59.3)	0.76	0.007	[9, 15, 17, 22, 26, 41, 45–47]
Male (%)	81.6 (56.3–100)	34	41.5 (21.2–67.3)	− 0.15	0.385	[9–15, 17, 18, 21, 22, 27, 29, 31, 32, 35, 36, 38–40, 43, 45–47, 60–64, 66–68, 70, 71]
Age at study	41.7 (34.9–50.5)	25	43.9 (21.2–67.3)	0.52	0.008	[9–11, 13–15, 17, 18, 21, 22, 27, 32, 36, 38–40, 42, 46, 47, 60, 61, 64, 66, 70, 71]
Age of onset	41.3 (34.9–50.5)	23	41.9 (25.0–67.3)	0.34	0.109	[10–12, 14, 15, 17, 18, 20–22, 31, 32, 36, 39–41, 43, 45–47, 60, 61, 70]
Smoking rate (%)	69.0 (41.0–87.0)	19	39.9 (25.0–67.3)	0.52	0.024	[9, 10, 12, 13, 17, 18, 22, 31, 32, 39, 40, 42, 46, 58, 60, 61, 66, 70, 71]
Drinking rate (%)	53.3 (6.5–90.6)	12	37.7 (25.0–67.3)	0.16	0.613	[9, 10, 12, 13, 17, 18, 22, 32, 50, 58, 60, 70]
**Attacks and bouts profile**						
Attack frequency	2.2 (1–5)	12	39.9 (25.0–55.7)	0.26	0.416	[11–13, 17, 18, 39, 40, 42, 46, 47, 61, 70]
Attack duration	94 (60–135.3)	12	39.9 (25.0–55.7)	0.08	0.811	[11–13, 17, 18, 39, 40, 42, 46, 47, 61, 70]
Bout Frequency	1.2 (0.4–1.9)	9	44.8 (25.0–55.7)	0.24	0.539	[12, 13, 17, 36, 39, 42, 46, 49, 61]
Bout Duration	6.5 (5.9–14.8)	14	48.1 (25.0–67.3)	0.07	0.812	[10, 12–14, 17, 18, 36, 39, 42, 46, 47, 56, 61, 70]
VAS (total 100)	87 (36–93)	12	37.6 (25.0–61.9)	− 0.48	0.116	[9, 11, 13–15, 17, 31, 32, 34, 48, 61, 70]
**Periodicity profiles (%)**						
Circannual rhythm	68.5 (47–83.3)	8	37.6 (25.0–67.3)	− 0.44	0.276	[10, 11, 17, 23, 31, 48, 57, 60]
Circadian rhythm	69.0 (52.9–83.3)	13	37.6 (25.0–67.3)	− 0.18	0.555	[10–12, 17, 18, 20, 23, 31, 32, 40, 58, 60, 61]
Nocturnal attacks	73.0 (58.0–83.3)	7	37.6 (25.0–67.3)	0.57	0.186	[9, 10, 17, 41, 60, 61, 71]
**Cranial autonomic symptoms (%)**						
Lacrimation	83.2 (52.8–100)	17	39.9 (25.0–67.3)	0.23	0.365	[9, 10, 12, 15, 17, 18, 31, 32, 34, 40–43, 47, 60, 61, 70]
Conjunctival injection	68.3 (37.4–95.7)	16	38.9 (25.0–59.3)	− 0.03	0.926	[9, 12, 15, 17, 18, 31, 32, 34, 40–43, 47, 60, 61, 70]
Nasal congestion	54.0 (23.33–100)	15	39.9 (25.0–59.3)	0.31	0.266	[9, 15, 17, 18, 31, 32, 34, 40–43, 47, 60, 61, 70]
rhinorrhea	58.0 (33.3–100)	16	38.9 (25.0–59.3)	0.12	0.661	[9, 12, 15, 17, 18, 31, 32, 34, 40–43, 47, 60, 61, 70]
Facial sweating	26.1 (4.3–57.7)	15	37.9 (25.0–67.3)	0.38	0.167	[9, 10, 12, 17, 18, 31, 32, 40, 41, 43, 47, 50, 60, 61, 70]
Eyelid edema	25.0 (0–75.0)	13	37.9 (25.0–55.7)	0.06	0.846	[9, 12, 17, 18, 31, 32, 34, 40, 41, 43, 47, 60, 61]
Ptosis	25.0 (8.5–83.6)	17	39.9 (25.0–67.3)	0.70	0.002	[9, 10, 12, 15, 17, 18, 31, 32, 34, 40–43, 47, 60, 61, 70]
Miosis	16.7 (2.4–49.6)	14	40.9 (25.0–67.3)	0.56	0.045	[10, 17, 18, 31, 32, 34, 40–43, 47, 60, 61, 70]
**Accompanying symptoms (%)**						
Nausea	42.1 (14.3–60.9)	12	37.1 (25.0–67.3)	− 0.69	0.012	[9, 10, 12, 14, 15, 17, 31, 32, 60, 61, 65, 70]
Vomiting	25.0 (7.0–38.5)	9	37.6(25.0–61.9)	− 0.59	0.095	[9, 12, 14, 17, 31, 32, 60, 61, 65]
Photophobia	44.1 (8.3–73.2)	13	37.9 (25.0–67.3)	0.46	0.115	[9, 10, 12, 14, 17, 31, 32, 39, 41, 60, 61, 65, 70]
Phonophobia	40.0 (8.0–73.2)	13	37.9 (25.0–67.3)	− 0.01	0.964	[9, 10, 12, 14, 17, 31, 32, 39, 41, 60, 61, 65, 70]
**Neuropsychiatric comorbidity (%)**						
Depression	13.1 (3.6–40.0)	7	44.8 (25.0–63.4)	0.50	0.258	[13, 38, 42, 71, 75, 77, 79]

The proportion of male patients showed a non-significant negative correlation with latitude (rho =  − 0.15, *p* = 0.385) (Table 2). The age in the study was significantly older in patients who dwelled in higher latitudes (rho = 0.519, *p* = 0.008), while the age at disease onset did not (rho = 0.34, *p* = 0.109). Though for every degree increase in latitude, the age in the study postponed by 0.19 years (*p* = 0.008), we did not find a correlation between diagnostic delay and latitude (rho = 0.19, *p* = 0.544). We also noted an increased trend of smoking rate among patients living in higher altitudes (rho = 0.52, p for rho = 0.024), and smoking rate increased by 0.5% for every degree increase of latitude (*p* = 0.027). There is no correlation between latitude and drinking rate (rho = 0.16, *p* = 0.613).

The latitude distribution of the 57 articles enrolled for clinical features ranged from 21.2° to 67.3° (median = 43.9°) (Table 2) There is no significant correlation between latitude and attack frequencies (rho = 0.26, *p* = 0.416), attack duration (rho = 0.08, *p* = 0.811), bout frequencies (rho = 0.24, *p* = 0.539), or bout duration (rho = 0.07, *p* = 0.812). For circadian and circannual rhythm and nocturnal attacks, the studies ranged from 25.0° to 67.3° (median = 37.6°). There is no significant correlation between latitude and circadian rhythm (rho =  − 0.18, *p* = 0.555), circannual rhythm (rho =  − 0.44, *p* = 0.276), or nocturnal attacks (rho = 0.57, *p* = 0.186).

For cranial autonomic symptoms, lacrimation remained the most commonly reported symptom (76.7%), and was followed by conjunctival injection (62.6%), rhinorrhea (56.6%), nasal congestion (48.5%), facial sweating (28.2%), eyelid edema (25.9%), ptosis (30.3%), and miosis (19.5%). Ptosis (rho = 0.70, *p* = 0.002) and miosis (rho = 0.56, *p* = 0.045) both showed significantly positive correlations with latitude. For every degree increase in latitude, there was a 1.3% increase in ptosis (*p* = 0.002) and a 0.7% increase in miosis (*p* = 0.045) (Table 2). The proportions of other symptoms, including lacrimation (rho = 0.23, *p* = 0.365), conjunctival injection (rho =  − 0.03, *p* = 0.926), nasal congestion (rho = 0.31, *p* = 0.266), rhinorrhea (rho = 0.12, *p* = 0.661), facial sweating (rho = 0.38, *p* = 0.167), and eyelid edema (rho = 0.06, *p* = 0.846), were not correlated with latitude.

Among the accompanying symptoms, nausea (rho =  − 0.69, *p* = 0.012) had a negative correlation with latitude. For every degree of increased latitude, the proportion of nausea decline by 0.7% (*p* = 0.012) (Table 2). Meanwhile, there is no significant correlation for vomiting (rho =  − 0.59, *p* = 0.095), photophobia (rho = 0.46, *p* = 0.115), or phonophobia (rho =  − 0.01, *p* = 0.964). We also did not find a correlation between the proportion of depression and latitude (rho = 0.50, *p* = 0.258).

For the factors that reached significant level on correlation analysis, after East–West category was adjusted, the significance for 1-year prevalence, proportion of cCH, smoking rate, ptosis, miosis, and nausea did not present for both latitude and East–West category (Suppl. Table 1).

## Discussion

In our study, we showed that (1) the 1-year prevalence, (2) smoking rate, (3) the proportion of cCH, either diagnosed by ICHD-3 alone or not, and (4) the proportion of miosis and/or ptosis were positively correlated with latitude. However, we did not discover a correlation between circadian rhythm, circannual rhythm, or the frequencies of nocturnal attacks with latitude. This study encompasses literature with populations located in tropical, temperate, and arctic zones and with latitudes spanning from 3.1° to 67.3°, providing several clinical insights into CH. To our knowledge, the correlation between CH prevalence and latitude has not been systematically investigated previously.

Currently, the actual prevalence of CH remains uncertain. In this study, we updated the median 1-year prevalence to be around 64/100,000 and the lifetime prevalence to be 99.6/10,000, which resembled the data from a widely accepted meta-analysis in 2008, which reported a 1-year prevalence of 53/100,000 and a lifetime prevalence 124/100,000 [82]. We noticed that both 1-year prevalence and lifetime prevalence had positive correlations with the latitude, though the trends were insignificant. However, after we removed a potential outlier, the correlation between latitude and 1-year prevalence became significant. For other primary headaches, data from migraine showed the lifetime prevalence ranging from 2.6 to 6.9% in six cities located between 0° to 25° and 12.5% to 26.5% in seven cities located higher than 30° latitude, which resembled our results [83]. The mechanism of how latitude affects the prevalence of CH was not well studied, and here, we proposed three plausible mechanisms, including (1) vitamin D deficiency, (2) the variation of total sunshine hours, and (3) the variation of temperature. First, vitamin D deficiency has been proposed as a major pathomechanism for latitude-related diseases such as multiple sclerosis, psychiatric disorders, and primary headache disorders [4, 83–85]. In a Korean study (Seoul, 37.53° N), though the rate of vitamin D deficiency was high for patients with CH (92.8%), this level was not significantly different from normal controls. Moreover, the level did not differ between cluster or remission periods [86]. In Scandinavian countries located in high latitudes, though exposed to less UVB radiation, the prevalence of vitamin D deficiency is lower than expected possibly due to a higher consumption of salmon and trout in their diets [87]. Thus, both studies showed that vitamin D deficiency could not fully explain the latitude-related prevalence of CH. Recently, genetic studies on CH have provided further evidence. In a recently published GWAS study, a single-nucleotide polymorphism (SNP) (rs12121134) near *DUSP10* was proposed to be related to CH, while this gene was also reported to be associated with vitamin D-related pathway [88, 89]. However, in a Greek genetic study, the authors examined three loci for the vitamin D receptor gene (*VDR*), and none of them showed a significant association with CH [90]. Another Swedish study examined a SNP (rs2228570) as in the Greek study also revealed negative results [91]. Taking the above findings together, the vitamin D pathway alone cannot fully explain the correlation between the prevalence of CH and latitudes. Second, it has been reported that the incidence of CH is inversely related to sunshine hours in a Taiwanese study [6, 83]. However, the total daylight hours in a year are distributed equally throughout the latitudes if the local weather conditions, such as cloudy days, were not taken into consideration [3, 92]. What is related to the latitude is the variation of sunlight [3]. Thus, for the Taiwanese study, the more appropriate explanation of the results might be due to the larger sunlight variation in Northern Taiwan [6]. Due to the scarce evidence on this topic, this hypothesis required further confirmation. A third explanation is the effect of temperature and its variation. A Greek study has shown that the average temperature is negatively correlated with both the latitude and the prevalence of daily headaches [93], though the study was not specific to CH. Moreover, another study reported that cluster attacks were more commonly presented during weather transitions, including seasonal and temperature changes [94]. Geographic evidence revealed a negative correlation between latitude and the absolute temperature, while a positive correlation was seen between the amplitude of temperature variation and latitude [3, 95, 96]. Taking the evidence together, we proposed that the larger temperature alteration, as well as the colder weather at higher latitudes, might be a factor that triggers CH. Since the hypothalamus is well-recognized as a center for temperature homeostasis and acts as a critical role in the pathomechanism of CH, how the climate at a higher latitude interacts with the function of the hypothalamus requires further clarification [2, 97].

It is now widely accepted that smoking is a risk factor for CH as well as the chronification of headaches, and their causal relationships were confirmed by a GWAS study utilizing Mendelian randomization [74]. We noticed that there was a higher smoking rate among patients in higher latitudes; however, this might be confounded by other factors since it is unreasonable to propose one’s smoking habit is affected by latitude. It has been reported that the smoking rates among Eastern studies (44.1% to 73.1%) were lower than the Western studies (67% to 88.3%), which resembled our results [72]. Since the Western studies selected in this study had a significantly higher latitude than the Eastern countries, after the adjustment was made, the significance of the effect of latitude disappeared. Thus, we proposed that the smoking rate remained largely affected by cultural or genetic differences between Eastern and Western populations [98].

Among the populations enrolled in this study, the proportion of cCH was significantly higher in those dwelled in higher latitudes, either the condition was diagnosed by all sorts of definition or by ICHD-3 alone. Several studies have suggested that smoking, a well-recognized risk factor for cCH, released cadmium and nicotine and disrupts the normally functioned hypothalamus-pituitary axis (HPA) [99, 100]. Moreover, though the hallmark of cCH is a loss of circannual periodicity or that bout lasted more than 9 months per year, we did not notice a longer bout duration or a loss of circadian presentation to be correlated with a higher latitude in this study. We believed that both environmental (smoking) and genetic factors concomitantly contribute to the chronification of CH. This hypothesis required further studies to examine their interactions.

Miosis and ptosis were both the results of sympathetic dysfunction, while lacrimation, conjunctival congestion, rhinorrhea, nasal congestions, or eyelid edema were due to activation of the parasympathetic reflex [101]. In this study, latitude correlated well with miosis and ptosis, but not with symptoms contributed by parasympathetic activation. We proposed two main mechanisms for this finding, including (1) the effect of temperature, and (2) the preserved hibernation habit. For the first hypothesis, previous studies showed that the prevalence of ptosis is correlated with age, diabetes, and high blood pressure and that the latter two diseases had inverse correlations with latitude, which might be the result of the lower ambient temperature at higher latitudes [102–104]. Some studies have suggested that miosis and ptosis were caused by the dysfunction of intracranial sympathetic nerves due to the dilatation of carotid arteries [105]. In an animal study, the carotid artery showed reversible vasodilatation by cooling from 37 to 4 ℃ to preserve cerebral blood flow [106]. These results could partially explain the higher prevalence of ptosis or miosis in colder places. For the second hypothesis, in animals dwelled at higher latitudes, they decreased their sympathetic tones and increased parasympathetic tones to prepare for hibernation during winter [107]. The results of the alteration of the autonomic nervous system during hibernation resembled the clinical features of CH and could to some degree echo the higher prevalence of CH in high-latitude residents. Both the hypotheses that a colder temperature and the adjustment of the autonomic nervous system during hibernation partly explained the higher presentation of miosis/ptosis at higher latitudes.

Depression has been reported to be more prevalent among CH patients than normal subjects [75]. However, we cannot find a significant relationship between the proportion of depression and latitude among CH patients. Our finding is in line with previous evidence that the relationship between depression and latitude was conflicting [108–110]. Due to the scarce data on CH patients, further investigation is required.

To sum up, we believe that the pathomechanism of CH remains multifactorial, which includes environmental, genetic, and bio-social factors. In this study, we have shown that latitude affects the prevalence of CH, the proportion of cCH, and symptoms related to sympathetic dysfunction. This might be due to the effect of environmental factors, such as the ambient temperature or sunlight exposure. Secondly, the discrepancies between Eastern and Western studies might have complicated our results [72]. Nevertheless, since the significant effect of latitude disappeared after adjusting for this factor in most variables, different genetic backgrounds among Eastern or Western populations remained a plausible explanation in this study. Lastly, it has been a widely acknowledged concept that smoking can cause CH or cCH [73, 74]. Though not widely explored in this study, smoking still represents an important bio-social factor in shaping the phenotypes of CH.

There are several limitations of this study. First, the number of studies enrolled for each variable was small, and therefore, this study is under-powered. However, we have done our best to explore as many studies as possible. Second, in each study enrolled, the methodology, the population included, the conducting periods, as well as the way to describe the clinical presentation differed, and these factors impede us from collecting a larger data more precisely. Third, for countries that span a wide range of latitudes i.e., the USA or China, though we assumed that the study population of a defined hospital were mostly residents near the location and had made our best to search for the enrolled population, still, conditions such as self-referral from a distant region could exist, especially in tertiary medical centers. Fourth, the enrolled study for clinical assessment encompassed only a small range of latitudes (21.2°–67.3°) for analysis, and the data of the countries in the tropical region and Asia remains lacking.

## Conclusions

This study showed that the 1-year prevalence, the proportion of chronic CH, as well as ptosis and mitosis were positively correlated with latitudinal change. Both temperature and sunlight variation at higher latitudes are plausible causes, and further studies are required for confirmation.

### Supplementary Information

Below is the link to the electronic supplementary material.

Supplementary file1 (DOCX 13 KB)

## Data Availability

Data is provided within the manuscript.
