# Supplementary material for: The Prevalence and Clinical Phenotypes of Cluster Headache in Relation with Latitude
Source: Curr Pain Headache Rep. 2024 Mar 5;28(5):427–38. doi: 10.1007/s11916-024-01229-3 (PMC11126473; doi:10.1007/s11916-024-01229-3)
Supplement: Supplementary file 1 — Supplementary file1 (DOCX 13 KB) [file 11916_2024_1229_MOESM1_ESM.docx]

**Supplement Table 1. Multivariable regression with latitude and East-West group**

| Items | P for latitude | P for E-W group | P for interaction |
| --- | --- | --- | --- |
| 1-year prevalence | 0.200 | 0.674 | 0.630 |
| cCH (%) | 0.885 | 0.517 | 0.906 |
| Smoking rate | 0.279 | 0.236 | 0.105 |
| Ptosis | 0.226 | 0.811 | 0.381 |
| Miosis | 0.648 | 0.940 | 0.609 |
| Nausea | 0.493 | 0.837 | 0.335 |
